# Pathological Internet use among European adolescents: psychopathology and self-destructive behaviours

**DOI:** 10.1007/s00787-014-0562-7

**Published:** 2014-06-03

**Authors:** Michael Kaess, Tony Durkee, Romuald Brunner, Vladimir Carli, Peter Parzer, Camilla Wasserman, Marco Sarchiapone, Christina Hoven, Alan Apter, Judit Balazs, Maria Balint, Julio Bobes, Renaud Cohen, Doina Cosman, Padraig Cotter, Gloria Fischer, Birgitta Floderus, Miriam Iosue, Christian Haring, Jean-Pierre Kahn, George J. Musa, Bogdan Nemes, Vita Postuvan, Franz Resch, Pilar A. Saiz, Merike Sisask, Avigal Snir, Airi Varnik, Janina Žiberna, Danuta Wasserman

**Affiliations:** 1 Section for Disorders of Personality Development, Clinic of Child and Adolescent Psychiatry, Centre for Psychosocial Medicine, University of Heidelberg, Heidelberg, Germany; 2 National Centre for Suicide Research and Prevention of Mental Ill-Health (NASP), Karolinska Institutet, Stockholm, Sweden; 3 Department of Child and Adolescent Psychiatry, New York State Psychiatric Institute, Columbia University, New York, NY USA; 4 Department of Health Sciences, University of Molise, Campobasso, Italy; 5 Department of Epidemiology, Mailman School of Public Health, Columbia University, New York, NY US; 6 Feinberg Child Study Centre, Schneider Children’s Medical Centre, Tel Aviv University, Tel Aviv, Israel; 7 Vadaskert Child and Adolescent Psychiatric Hospital, Budapest, Hungary; 8 Institute of Psychology, Eotvos Lorand University, Budapest, Hungary; 9 Pedagogical and Psychological Counsel, Kobanya, Hungary; 10 Department of Psychiatry, School of Medicine, Centro de Investigación Biomédica en Red de Salud Mental CIBERSAM, University of Oviedo, Oviedo, Spain; 11 Department of Psychiatry, Centre Hospitalo-Universitaire de NANCY, Université H. Poincaré, Nancy, France; 12 Department of Clinical Psychology Department, Iuliu Hatieganu University of Medicine and Pharmacy, Cluj-Napoca, Romania; 13 National Suicide Research Foundation, Cork, Ireland; 14 Department of Clinical Neuroscience, Karolinska Institutet, Stockholm, Sweden; 15 Research Division for Mental Health, University for Medical Information Technology (UMIT), Innsbruck, Austria; 16 Health Research Department, PINT, University of Primorska, Koper, Slovenia; 17 Estonian-Swedish Mental Health and Suicidology Institute, Centre of Behavioural and Health Science, Tallinn University, Tallinn, Estonia

**Keywords:** Pathological Internet use, Internet addiction, Internet gaming disorder, SEYLE, Psychopathology, Suicidal behaviour

## Abstract

Rising global rates of pathological Internet use (PIU) and related psychological impairments have gained considerable attention in recent years. In an effort to acquire evidence-based knowledge of this relationship, the main objective of this study was to investigate the association between PIU, psychopathology and self-destructive behaviours among school-based adolescents in eleven European countries. This cross-sectional study was implemented within the framework of the European Union project: Saving and Empowering Young Lives in Europe. A representative sample of 11,356 school-based adolescents (M/F: 4,856/6,500; mean age: 14.9) was included in the analyses. PIU was assessed using the Young’s Diagnostic Questionnaire. Psychopathology was measured using the Beck Depression Inventory-II, Zung Self-Rating Anxiety Scale and Strengths and Difficulties Questionnaire. Self-destructive behaviours were evaluated by the Deliberate Self-Harm Inventory and Paykel Suicide Scale. Results showed that suicidal behaviours (suicidal ideation and suicide attempts), depression, anxiety, conduct problems and hyperactivity/inattention were significant and independent predictors of PIU. The correlation between PIU, conduct problems and hyperactivity/inattention was stronger among females, while the link between PIU and symptoms of depression, anxiety and peer relationship problems was stronger among males. The association between PIU, psychopathology and self-destructive behaviours was stronger in countries with a higher prevalence of PIU and suicide rates. These findings ascertain that psychopathology and suicidal behaviours are strongly related to PIU. This association is significantly influenced by gender and country suggesting socio-cultural influences. At the clinical and public health levels, targeting PIU among adolescents in the early stages could potentially lead to improvements of psychological well-being and a reduction of suicidal behaviours.

**Electronic supplementary material:**

The online version of this article (doi:10.1007/s00787-014-0562-7) contains supplementary material, which is available to authorized users.

## Introduction

The Internet is an integral part of modern society and functions as an essential medium for communication, socialization and education. Global rates of Internet use have increased considerably over the past few decades [1]. Though this trend is observed in all age groups, it is most pronounced among youth [2]. The expansion of interactive online activities appears to incite youth to stay online longer than anticipated [3, 4]. Excessive use of the Internet is known to increase the propensity for developing online addictive behaviours [5, 6]. These high risk-behaviours could have a detrimental impact on adolescents’ psychological development [7, 8].

Pathological Internet use (PIU) is conceptualized as an impulse-control disorder [9] and regarded as a taxonomy of behavioural addiction [10]. There is accumulating scientific-based evidence showing that PIU shares psychosocial, neurological, biological and genetic physiognomies with both pathological gambling and substance use disorders [11–16] with particular findings within Internet gaming [17, 18]. In the recently published Diagnostic and Statistical Manual of Mental Disorders fifth edition (DSM-5), Internet gaming disorder was incorporated into Section III stipulating that further research was required [19]. In the new edition of the International Classification of Diseases (ICD-11), the WHO task force is considering the integration of behavioural addiction as a new diagnostic category with computer and Internet addiction as a subcategory [20]. The changes in nomenclature are potentially due to the mounting evidence suggesting PIU is associated with severe psychological impairment, particularly among youth [21, 22].

There are several cross-sectional studies indicating notable associations between PIU and symptoms of depression [23, 24], anxiety [25, 26], attention deficit hyperactivity disorder (ADHD) [27, 28], obsessive–compulsive disorder [29] and hostility/aggression [30]. Recent systematic reviews, however, have underlined a gap in knowledge due to methodological discrepancies and insufficient data concerning the relationship between PIU and comorbid psychopathology [31, 32].

Research shows that self-destructive behaviours are frequently observed among adolescents. These behaviours are typically interconnected with comorbid psychopathology [33, 34] and various types of risk-behaviours [35, 36]; however, little attention has been given to its potential link with PIU. The existing literature comprises limited evidence specifically assessing the liaison between PIU and self-destructive behaviours. Despite the ambiguity of research, there are a few studies indicating correlations between PIU and self-injurious behaviours (SIB) [37], suicidal ideation [38–40] and suicide attempts [41]. There are currently no studies identified in the literature examining this relationship using a cross-national European sample.

Given the limited data, studies using large and homogenous samples with reliable and validated psychometric measures are necessitated in order to assess the complex interaction between PIU, psychopathology and self-destructive behaviours. Moreover, determining the influence of gender and country in this relationship will improve our understanding of the condition.

The present study is performed on a large, representative, cross-national sample of school-based adolescents in eleven European countries. The main objectives of this study were: (1) to investigate the association between PIU and psychopathological symptoms of depression, anxiety, emotional problems, conduct problems, hyperactivity/inattention and peer relationship problems; (2) to examine the association between PIU and self-destructive behaviours including SIB, suicidal ideation and suicide attempts; and (3) to assess the influence of gender and country on the relationship between PIU, psychopathology and self-destructive behaviours.

## Methods

### Study design and recruitment

The present cross-sectional study was performed within the framework of the European Union project: Saving and Empowering Young Lives in Europe (SEYLE) [42]. This randomized controlled trial (RCT) was implemented in eleven countries: Austria, Estonia, France, Germany, Hungary, Ireland, Israel, Italy, Romania, Slovenia and Spain with Sweden serving as the coordinating centre. The SEYLE sample comprised 12,395 school-based adolescents. Adolescents were recruited from randomly selected schools (*n* = 179) across study sites in each country according to specific inclusion and exclusion criteria [42]. Ethical approval was obtained from the local ethical committees in each respective country. Consent, response rates and representativeness of the sample were scrutinized and reported in a methodological analysis [43]. The analysis showed that study sites were reasonably representative of their respective country indicating a high external validity. Out of the initial sample of 12,395 students who completed the baseline questionnaire, 1,039 students were excluded due to missing data relevant for this study. The final sample comprised 11,356 school-based adolescents for the present study.

### Measurements

Pathological Internet use (PIU) was assessed using the Young’s Diagnostic Questionnaire (YDQ) [44]. The 8-item questionnaire has been found to be a reliable instrument for ascertaining PIU among adolescents [45]. The YDQ assesses patterns of Internet usage that result in psychological or social distress. The 8-item score reflects eight of the nine criteria for Internet gaming disorder in the DSM-5; however, the YDQ allows for the assessment of all online activities. Based on the YDQ total score, Internet users were categorized into three groups: adaptive Internet users (AIU) (scoring 0–2); maladaptive Internet users (MIU) (scoring 3–4); and pathological Internet users (PIU) (scoring ≥ 5) [46].

The Beck Depression Inventory-II (BDI-II) [47] is a 21-item questionnaire used to measure depressive symptoms. A modified version of the BDI-II was used in the present study. One item “loss of libido” was removed from the scale as it was considered in some countries to be an inappropriate question for an adolescent population. Evidence shows that the omission of this question does not affect the reliability or validity of the BDI-II [48]. Our study confirmed this by showing a Cronbach’s alpha coefficient of (*α* = 0.86) [43]. Based on the BDI-II total score, students were categorized as having no depression (scoring 0–13), mild depression (scoring 14–19), moderate depression (scoring 20–28) and severe depression (scoring 29–63).

Anxiety was measured by the Zung Self-Rating Anxiety Scale (Z-SAS) [49]. Z-SAS is a 20-item assessment scale that measures state and trait anxiety. Based on the Z-SAS total score, adolescents were categorized as normal (scoring ≤ 44), minimal to moderate anxiety (scoring 45–59), severe anxiety (scoring 60–74) and extreme anxiety (scoring ≥ 75).

The Strengths and Difficulties Questionnaire (SDQ) [50] is a 25-item measure used for adolescents aged 11–17 years. The SDQ assesses emotional symptoms, conduct problems, hyperactivity/inattention, peer relationship problems and pro-social behaviour. The score is generated by summing items from all subscales. Based on the SDQ total score, students were categorized as normal (scoring 0–15), borderline (scoring 16–19) and severe (scoring 20–40).

A modified version of the Deliberate Self-Harm Inventory (DSHI) [51] was utilized to measure SIB. In the present study, different methods of SIB were combined to simplify and shorten the measure to a 6-item questionnaire [52]. SIB was rated positive in cases reporting ≥5 events of SIB during their lifetime.

Suicidal behaviours (suicidal ideation and suicide attempts) were measured by the Paykel Suicide Scale (PSS) [53]. The PSS comprises the following five questions: during the past 2 weeks have you (i) *felt that life was not worth living*; (ii) *wished you were dead*; (iii) *thought of taking your own life*; (iv) *seriously considered taking your own life or even made plans;* and (v) *have you tried to take your own life*? A category of suicidal thoughts was defined by the answer “Yes” to the third (iii) and fourth (iv) question of the PSS (PSS score of 3–4). Suicide attempts were defined by the answer “Yes” to the last question (v) of the PSS (PSS score of 5).

### Statistical analyses

The prevalence of categories in the BDI-II, Z-SAS, SDQ, DSHI and PSS was calculated independently for each Internet user group. To analyse the relationship between PIU (as the YDQ score), psychopathology and self destructive behaviours, multilevel mixed-effects linear regression with PIU as the dependent variable and age, gender, psychopathological scores (BDI-II, Z-SAS, SDQ) and categorical variables of self-destructive behaviours (DSHI, PSS) as level 1 fixed effects, school as level 2 random intercept and country as level 3 random intercept was performed. The estimation method was full ML with independent covariance structure. A subsequent stepwise reduction of the regression model was conducted in order to minimize the Bayes information criterion (BIC). We also explored possible interactions of the predictors with gender and country. Including all these interactions in one model would render a complex model with several estimation problems. Therefore, we analysed each predictor in a separate model with PIU as the dependent variable, the predictor, gender, country, interaction of predictor with gender and interaction of the predictor with country as level 1 fixed effects and school as level 2 random intercept. In the aforementioned, the estimation method was full ML with independent covariance structure. To avoid estimation problems due to cells with zeroes, we had to combine the categories “suicidal thoughts” and “suicide attempts” into one category “suicidal behaviours”. All calculations were performed using the statistical software Stata 13.

## Results

### Characteristics of the sample

The final study sample comprised 11,356 adolescent students: 4,856 male (42.8 %) and 6,500 female (57.2 %) with a mean age of 14.9 years (SD ± 0.88). The prevalence of MIU and PIU was 13.4 and 4.2 %, respectively. Females reported higher MIU (14.2 %) compared to males (12.3 %), while PIU was slightly higher among males (4.7 %) compared to females (3.9 %).

### Psychopathology and PIU

In the AIU group (*n* = 9,355), results showed that the prevalence of moderate to severe depression and anxiety was 5 %, respectively. In the MIU group (*n* = 1,523), 17.1 % were identified with moderate to severe depression and 16.4 % with moderate to severe anxiety. In the PIU group (*n* = 478), 33.5 % reported moderate to severe depression and 27.6 % reported moderate to severe anxiety. The proportion of depression and anxiety was significantly higher in the MIU and PIU groups.

The prevalence of borderline and severe emotional symptoms was 10.8 % for adaptive users, 23.7 % for maladaptive users and 32 % for pathological users. This pattern continued for the prevalence of conduct problems (17.5, 31.9, 41.0 %), hyperactivity/inattention (15.1, 28.2, 37.2 %), peer relationships (12.6, 21.6, 31.2 %) and low pro-social behaviours (14.1, 18.1, 25.1 %), respectively. The proportion of all borderline and severe categories was significantly higher in the MIU and PIU groups (see Supplement Table A for prevalence rates and statistics on group differences).

### Self-destructive behaviours and PIU

The prevalence of SIB was 4.5 % among adaptive users, 12.2 % among maladaptive users and 22.2 % among pathological users. These outcomes denote that SIB was nearly three times higher among maladaptive users and nearly five times higher among pathological users compared to adaptive users. A similar pattern was observed for the prevalence of suicidal ideation (12.7, 31.9, 42.3 %) and suicide attempts (0.3, 1.1, 3.1 %). The proportion of suicidal ideation was two and a half times higher among maladaptive users and three times higher among pathological users compared to adaptive users, while suicide attempts were nearly four times higher among maladaptive users and ten times higher among pathological users compared to adaptive users (see Supplement Table A).

### The association between PIU and age, gender, psychopathology and self-destructive behaviours

Results from the multivariate regression analysis on age, gender, psychopathology and self-destructive behaviours among pathological users are presented in Table 1. Outcomes indicated a significant inverse correlation between female gender and PIU (*p* < 0.001). The strongest correlations within the psychopathological domain were observed between PIU and symptoms of depression (*p* < 0.001) and anxiety (*p* < 0.001). In the SDQ, notable correlations were observed between PIU and conduct problems (*p* < 0.001) and hyperactivity/inattention (*p* < 0.001); however, no significant associations were found between PIU and emotional symptoms, peer relationship problems or low pro-social behaviours. In regards to self-destructive behaviours, SIB (*p* = 0.014), suicidal ideation (*p* < 0.001) and suicide attempts (*p* = 0.003) were significantly correlated with PIU.

**Table 1:** Multivariate regression model on pathological Internet use (PIU) with age, gender, psychopathological scores and self-destructive behaviour categories as explaining variables

Psychopathology and self-destructive behaviours	Pathological Internet use			
	Coefficient	95 % CI	Standardized coefficient^a^	*p* value
Age	−0.014	−0.048 to 0.020	−0.009	0.426
Female gender	−0.086	−0.141 to −0.032	–	0.002
Depression (BDI-score)	0.032	0.027 to 0.037	0.166	<0.001
Anxiety (Z-SAS-score)	0.033	0.028 to 0.038	0.169	<0.001
Emotional symptoms (SDQ-subscore)	−0.002	−0.017 to 0.013	−0.003	0.825
Conduct problems (SDQ-subscore)	0.059	0.041 to 0.077	0.064	<0.001
Hyperactivity and/or inattention (SDQ-subscore)	0.057	0.044 to 0.070	0.084	<0.001
Peer relationship problems (SDQ-subscore)	−0.007	−0.024 to 0.009	−0.008	0.375
Pro-social behaviour (SDQ-subscore)	−0.011	−0.026 to 0.003	−0.015	0.121
Self-injurious behaviour	0.132	0.027 to 0.237	–	0.014
Suicidal ideation	0.314	0.241 to 0.387	–	<0.001
Suicide attempts	0.530	0.185 to 0.875	–	0.003
Random-effects parameters				
Country (variance of intercept)	0.044	0.177 to 0.111		
School (variance of intercept)	0.032	0.022 to 0.049		

Young’s Diagnostic Questionnaire (YDQ)

Model Wald *χ* ^2^(12) = 2,605.9, *p* < 0.0001; LR test versus linear regression *χ* ^2^(2) = 349.1, *p* < 0.0001, BIC = 37,976,94

^a^The standardized coefficient of continuous predictor *x* was calculated as coef(*x*) × SD(*x*)/SD(*y*)

The stepwise regression analysis resulted in a model that showed gender, symptoms of depression, anxiety, conduct problems, hyperactivity/inattention, suicidal ideation and suicide attempts were significant and independent predictors of PIU. SIB did not appear to be a significant factor in the final model (see Table 2).

**Table 2:** Final model of the stepwise regression on pathological Internet use (PIU) with age, gender, psychopathological scores and suicidal behaviour categories as explaining variables

Psychopathology and self-destructive behaviours	Pathological Internet use			
	Coefficient	95 % CI	Standardized coefficient^a^	*p* value
Female gender	−0.971	−0.149 to −0.045		<0.001
Depression (BDI-score)	0.033	0.028 to 0.038	0.168	<0.001
Anxiety (Z-SAS-score)	0.033	0.028 to 0.038	0.169	<0.001
Conduct problems (SDQ-subscore)	0.063	0.045 to 0.080	0.068	<0.001
Hyperactivity and/or inattention (SDQ-subscore)	0.059	0.046 to 0.071	0.086	<0.001
Suicidal ideation	0.324	0.251 to 0.397		<0.001
Suicide attempts	0.552	0.207 to 0.896		0.002
Random-effects parameters				
Country (variance of intercept)	0.043	0.172 to 0.108		
School (variance of intercept)	0.033	0.022 to 0.050		

Young’s Diagnostic Questionnaire (YDQ)

Model Wald *χ* ^2^(7) = 2,593.2, *p* < 0.0001; LR test versus linear regression *χ* ^2^(2) = 357.96.1, *p* < 0.0001, BIC = 37,940.35

^a^The standardized coefficient of continuous predictor x was calculated as coef(*x*) × SD(*x*)/SD(*y*)

### Interactions of psychopathology and self-destructive behaviours with gender and country

The effect of gender and country on the relationship between PIU, psychopathology and self-destructive behaviours is presented in Table 3.

**Table 3:** Interactions of psychopathology and self-destructive behaviour with gender and country in the regression analysis of psychopathological scores and self-destructive behaviour categories on pathological Internet use (PIU)

Psychopathology and self-destructive behaviours	Interactions with gender		Gender with stronger association between variables and YDQ-score	Interactions with country		Country with stronger association between variable and YDQ-score	Country with weaker association between variable and YDQ-score
Variables	*χ* ^2^ (df = 1)	*p* value		*χ* ^2^ (df = 10)	*p* value		
Depression (BDI-score)	19.28	<0.001	Male	85.52	<0.001	Ireland	Italy
Anxiety (Z-SAS score)	10.53	0.001	Male	49.54	<0.001	Estonia	France
Emotional symptoms (SDQ-subscore)	1.89	0.169	–	47.52	<0.001	Estonia	Hungary
Conduct problems (SDQ-subscore)	6.26	0.012	Female	28.74	0.001	Ireland	Italy
Hyperactivity and/or inattention (SDQ-subscore)	13.62	<0.001	Female	50.59	<0.001	Slovenia	Israel
Peer relationship problems (SDQ-subscore)	8.16	0.004	Male	19.17	0.038	Ireland	France
Pro-social behaviour (SDQ-subscore)	3.04	0.081	–	10.07	0.435	–	–
Self-injurious behaviour (Category of 5 or more incidents of self-harm in the DSHI)	0.05	0.831	–	46.75	<0.001	Ireland	Slovenia
Suicidal behaviour (Category of suicidal thoughts and suicide attempts in the PSS)	0.35	0.553	–	90.19	<0.001	Estonia	Italy

Young’s Diagnostic Questionnaire (YDQ)

Results showed that gender had a significant effect on the relationship between PIU and symptoms of depression, anxiety, conduct problems, hyperactivity/inattention and peer relationship problems. The correlation between PIU and symptoms of depression, anxiety and peer relationship problems was stronger among males, whereas the link between conduct problems, hyperactivity/inattention and PIU was stronger among females. No significant gender interactions were found with regards to self-destructive behaviours.

Significant cross-national interactions showed that country markedly influenced the relationship between PIU, psychopathology and self-destructive behaviours. The correlation between PIU, psychopathology and self-destructive behaviours was strongest in Estonia and Ireland, while the weakest correlations were observed in France, Italy and Hungary.

## Discussion

Pathological Internet use (PIU) appears to be strongly correlated with comorbid psychopathology and suicidal behaviours. Results revealed that the prevalence of psychopathology and self-destructive behaviours was higher among pathological users compared to both adaptive and maladaptive users. Given addiction is a chronic and progressive disease [54], identifying and treating individuals in the earlier stages can theoretically prevent further escalation into a fully developed addiction, psychopathological disorder or suicidal behaviour. In this context, tracing the development of maladaptive patterns of Internet use provides the opportunity to intervene before these behaviours become pathological.

In the final stepwise regression analysis, results showed that symptoms of depression, anxiety, conduct problems, hyperactivity/inattention and suicidal behaviours were significant and independent predictors of PIU. If adolescents have pre-existing psychopathological symptoms, then the Internet could serve as means to avoid or escape from psychological distress. Coping strategies are an effort to solve personal and interpersonal problems in order to escape, avoid or minimize stress levels [55]. Thompson et al. [56] suggest that there are two types of coping strategies involved in depressive states: (i) adaptive coping (i.e., strategies that reduce stress levels) and (ii) maladaptive coping (i.e., strategies that induce stress levels). Yen and colleagues [57] noted that the level of hostility and depression among subjects markedly decreased as soon as they went online. This outcome infers that Internet use could develop into a maladaptive coping strategy. On the other hand, it could be hypothesized that students exhibit precipitating symptoms of psychopathology or suicidal behaviours as a result of their online experiences. Research examining the pathway of this relationship is needed. The role of psychopathology and suicidal behaviours at different stages of Internet use is important to understand. If mental disorders are treated in the earlier stages, this is known to delay or prevent subsequent suicidal acts [58]. The same reasoning could apply to targeting early symptoms of PIU among adolescents.

Adolescents with conduct problems and hyperactivity/inattention are often impulsive [59, 60]. Impulsivity is an important trait in the context of suicidal behaviours [61, 62]. As PIU is considered to be an impulse-control disorder, conditions associated with impulsivity are expected to be present among pathological users, which is clearly supported by our findings. In this regard, PIU seems to show considerable similarities with substance use to which there is strong evidence showing significant correlations with psychopathology, suicidal behaviours and impulsivity [63, 64]. A recent review argued that certain traits as impulsivity are potential endophenotypes of a variety of mental health problems [65]. These underlying character traits could be essential in the development of PIU and may explain the robust associations observed between PIU and impulsive-related emotional and behavioural problems and suicidal behaviours in the present study. Further investigations on PIU and personality traits could help to characterize and identify at-risk groups in the early stages of addiction in order to facilitate prevention efforts.

Our results showed that male students experiencing symptoms of depression, anxiety and peer relationship problems were more likely to be identified as pathological users; however, these symptoms are known to occur more often in females [66, 67]. Female students experiencing conduct problems or hyperactivity/inattention were more likely to be identified as pathological users; however, these behaviours are known to be more prevalent among males [68, 69]. These outcomes infer that suffering from atypical gender-specific problems appears to be significantly associated with PIU. It could be postulated that males and females exhibit different pathways in the development of PIU.

Our findings revealed that country significantly influenced the association between PIU, psychopathology and self-destructive behaviours. The correlation between PIU, psychopathology and self-destructive behaviours was strongest in Estonia and Ireland, while the weakest was found in France, Italy and Hungary. This corresponds with the prevalence of PIU in these respective countries [70]. The observed trend also corresponds to the national suicide rates among these respective countries. In 2010, suicide rates were higher among those aged 15–19 years in Estonia (12.8 %) and Ireland (10.8 %) compared to adolescents in France (4.9 %), Italy (1.9 %) and Hungary (5.9 %) [71]. These results infer that country could be a moderating factor in the correlation between PIU, psychopathology and self-destructive behaviours. The moderating role of country could be due to genetic variability or the notable effect of socio-cultural influences. Socio-cultural factors as cultural identity, family structure and gender roles are known to affect adolescent behaviours [72, 73] and psychological well-being [74]. Online addictive behaviours and related psychopathological features observed among adolescents could be a reflection of the internalized values and beliefs expressed in their respective country. Research explicitly assessing the moderating role of intercultural influences in the development of PIU and psychopathology is still lacking and necessitates further investigations.

To our knowledge, this is the first epidemiological study to investigate the association between PIU, psychopathology and self-destructive behaviours within a cross-national European context. Research in this field has been hampered by methodological weaknesses, whereof the most prominent has been sampling bias. The majority of previous studies frequently relied on convenience sampling through voluntary Internet surveys (e.g., chat rooms) without measurable denominators. The novelty of this study is the large, representative, cross-national sample of European adolescents. The standardized and homogenous methodology used to collect the data is also a major strength of the study. Given the study sites were representative of the respective country allows for a higher degree of generalizability of the findings. Limitations of the study included the cross-sectional design, which provides no indication of the sequence of events and is unable to infer causality. Data were collected through self-reported questionnaires, which are susceptible to information bias.

## Conclusions

Results showed that symptoms of depression, hyperactivity/inattention, conduct problems and suicidal behaviours are significant and independent predictors of PIU. Suicidal behaviours, depression and anxiety proved to be the strongest predictors of PIU. This interaction is significantly influenced by gender and country suggesting socio-cultural influences. At the clinical and public health levels, targeting PIU among adolescents in the early stages could potentially lead to improvements of psychological well-being and a reduction of suicidal behaviours.

## Electronic supplementary material

Below is the link to the electronic supplementary material.

Supplementary material 1 (DOCX 50 kb)
