# Supplementary material for: Pathological Internet use among European adolescents: psychopathology and self-destructive behaviours
Source: Eur Child Adolesc Psychiatry. 2014 Jun 3;23(11):1093–102. doi: 10.1007/s00787-014-0562-7 (PMC4229646; doi:10.1007/s00787-014-0562-7)
Supplement: Supplementary file 1 — Supplementary material 1 (DOCX 50 kb) [file 787_2014_562_MOESM1_ESM.docx]

**Supplement**

**Supplement Table A:** Prevalence of psychopathology among adolescents by Internet user group

| **Psychopathology** | **Adaptive use** | | **Maladaptive use (MIU)** | | **Pathological use (PIU)** | | **Total** | |
| --- | --- | --- | --- | --- | --- | --- | --- | --- |
|  | n=9355 | % | n=1523 | % | n=478 | % | N=11356 | % |
|  |  |  |  |  |  |  |  |  |
| **Depression** | Group differences between Internet user groups: χ^2^(6)=1000.00; p<0.001; τ_b_=0.27 | | | | | | | |
| No | 8137 | 86.98 | 975 | 64.02 | 224 | 46.86 | 9336 | 82.21 |
| Mild | 738 | 7.89 | 288 | 18.91 | 94 | 19.67 | 1120 | 9.86 |
| Moderate | 355 | 3.79 | 189 | 12.41 | 102 | 21.34 | 646 | 5.69 |
| Severe | 125 | 1.34 | 71 | 4.66 | 58 | 12.13 | 254 | 2.24 |
| **Anxiety** | Group differences between Internet user groups: χ^2^(4)=600.88; p<0.001; τ_b_=0.21 | | | | | | | |
| Normal | 8884 | 94.97 | 1273 | 83.59 | 346 | 72.38 | 10503 | 92.49 |
| Moderate | 450 | 4.81 | 234 | 15.36 | 111 | 23.22 | 795 | 7.00 |
| Severe | 21 | 0.22 | 16 | 1.05 | 21 | 4.39 | 58 | 0.51 |
| **Emotional symptoms** | Group differences between Internet user groups: χ^2^(4)=343.00; p<0.001; τ_b_=0.16 | | | | | | | |
| Normal | 8342 | 89.17 | 1162 | 76.30 | 325 | 67.99 | 9829 | 86.55 |
| Borderline | 459 | 4.91 | 157 | 10.31 | 56 | 11.72 | 672 | 5.92 |
| Severe | 554 | 5.92 | 204 | 13.39 | 97 | 20.29 | 855 | 7.53 |
| **Conduct problems** | Group differences between Internet user groups: χ^2^(4)=356.91; p<0.001; τ_b_=0.16 | | | | | | | |
| Normal | 7721 | 82.53 | 1038 | 68.15 | 282 | 59.00 | 9041 | 79.61 |
| Borderline | 921 | 9.85 | 213 | 13.99 | 76 | 15.90 | 1210 | 10.66 |
| Severe | 713 | 7.62 | 272 | 17.86 | 120 | 25.10 | 1105 | 9.73 |
| **Hyperactivity and/or inattention** | Group differences between Internet user groups: χ^2^(4)=299.41; p<0.001; τ_b_=0.15 | | | | | | | |
| Normal | 7946 | 84.94 | 1093 | 71.77 | 300 | 62.76 | 9339 | 82.24 |
| Borderline | 700 | 7.48 | 187 | 12.28 | 72 | 15.06 | 959 | 8.44 |
| Severe | 709 | 7.58 | 243 | 15.96 | 106 | 22.18 | 1058 | 9.32 |
| **Peer relationship problems** | Group differences between Internet user groups: χ^2^(4)=211.48; p<0.001; τ_b_=0.12 | | | | | | | |
| Normal | 8173 | 87.37 | 1194 | 78.40 | 329 | 68.83 | 9696 | 85.38 |
| Borderline | 957 | 10.23 | 243 | 15.96 | 108 | 22.59 | 1308 | 11.52 |
| Severe | 225 | 2.41 | 86 | 5.65 | 41 | 8.58 | 352 | 3.10 |
| **Pro-social behaviour** | Group differences between Internet user groups: χ^2^(4)=66.90; p<0.001; τ_b_=0.06 | | | | | | | |
| Normal | 8036 | 85.90 | 1248 | 81.94 | 358 | 74.90 | 9642 | 84.91 |
| Borderline | 775 | 8.28 | 141 | 9.26 | 58 | 12.13 | 974 | 8.58 |
| Severe | 544 | 5.82 | 134 | 8.80 | 62 | 12.97 | 740 | 6.52 |
| **Self-injurious behaviour** | Group differences between Internet user groups: χ^2^(2)=348.83; p<0.001; τ_b_=0.16 | | | | | | | |
| No | 8936 | 95.52 | 1337 | 87.79 | 372 | 77.82 | 10645 | 93.74 |
| Yes | 419 | 4.48 | 186 | 12.21 | 106 | 22.18 | 711 | 6.26 |
| **Suicidal ideation/behaviour** | Group differences between Internet user groups: χ^2^(4)=694.67; p<0.001; τ_b_=0.23 | | | | | | | |
| None | 8140 | 87,01 | 1020 | 66.98 | 261 | 54.60 | 9421 | 82.96 |
| Suicidal ideation | 1192 | 12.74 | 486 | 31.91 | 202 | 42.26 | 1880 | 16.55 |
| Suicide attempts | 23 | 0.25 | 17 | 1.12 | 15 | 3.14 | 55 | 0.48 |

τ_b_= Kendall's Tau b
